# Investigation of insulin-like growth factors/insulin-like growth factor binding proteins regulation in metabolic syndrome patients

**DOI:** 10.1186/s13104-019-4492-9

**Published:** 2019-10-11

**Authors:** Somayeh Pouriamehr, Haleh Barmaki, Mozhdeh Rastegary, Farzaneh Lotfi, Mohsen Nabi Afjadi

**Affiliations:** 1 Department of Biology, Dezful Branch, Islamic Azad University, Dezful, Iran; 2 Department of Laboratory Medicine, Faculty of Para Medical Sciences, Shahid Behest University of Medical Sciences, Tehran, Iran; 3 Department of Biology, Basic Science Faculty, Shahrekord Branch, Islamic Azad University, Shahrekord, Iran; 4 Department of Clinical Biochemistry, Faculty of Medicine, Kermanshah University of Medical Sciences, Kermanshah, Iran; 5 Institute of Biochemistry and Biophysics, Tehran, Iran

**Keywords:** Metabolic syndrome X, Insulin-like growth factor, Insulin-like growth factor receptor

## Abstract

**Objective:**

The insulin-like growth factors (IGFs) and their binding proteins (IGFBPs) are thought to play a significant role in metabolic pathways and glucose metabolism. Unregulated levels of IGFs/IGFBPs have been associated with the development of glucose intolerance and metabolic syndrome X (MSx). We hypothesized that change of IGFs/IGFBPs levels could increase the risk of MSx; thus, this study aimed to evaluate the serostatus of IGFs/IGFBPs in individuals with MSx.

**Results:**

After adjustment for metabolic parameters, MSx patients had a lower level of IGF-1, IGFBP-1, and IGFBP-2 compared with subjects in the control group. Further analysis revealed a positive correlation between serum levels of IGF-1 and IGF-2 (p < 0.05), as well as serum IGFBP-3 and IGF-2 (p < 0.05). Also, the statistical analysis showed a negative association of serum IGF-1 with plasma glucose and total cholesterol levels (p < 0.05). Besides, a negative relationship was found between serum concentrations of IGF-1/IGF-2 and the risk of developing MSx. These data indicated that some components of IGFs/IGFBPs are linked with the pathogenesis of MSx. In conclusion, these inverse associations showed a possible linkage between the IGF/IGFBP signaling pathway and the development of MSx. It seems the decreased concentrations of IGFs edmay be regarded as a potential biomarker for early diagnosis or even prognosis of MSx but need more systematic studies to confirmed it.

## Introduction

Metabolic syndrome X (MSx) is a growing health burden, and it has been associated with an increased risk of diabetes and coronary heart disease [1]. Several risk factors have been ascribed to the development of MSx, such as central obesity, low levels of high-density lipoprotein (HDL-c) cholesterol, hypertriglyceridemia, hypertension, and impaired blood glucose regulation [1–3]. Insulin-like growth factors (IGFs) are considered the major components of regulatory mediators of the growth process. Studies have demonstrated that IGFs and insulin-like growth factor binding proteins (IGFBPs) contribute to other critical metabolic pathways, namely IGF/insulin-dependent pathway and IGF/insulin-independent pathway [4, 5]. Previous reports have shown that the elements of the IGF/IGFBP system have vital roles in the modulation of insulin and glucose metabolism [4, 6]. Recently, it has been indicated that IGFBPs have a significant impact on the metabolic-related functions as they play a crucial role in the pathogenesis of obesity, insulin resistance, and development of MSx [4, 5]. Previous studies indicated that dysregulated levels of IGF-1 could cause impairment in glucose tolerance. For instance, a decrease in IGFBP1 production leads to a reduction in IGF-1 levels, and it is capable of inducing hyperinsulinemia and abnormal glucose clearance [7]. Also, IGF-2 inhibits hepatic glucose synthesis and prevents glycogen production. In this context, the interaction between IGFs and their cognate IGFBPs can antagonize their biological activity, resulting in the induction of hypoglycemia [8]. Numerous research has shown that obesity is linked with the development of insulin resistance, hyperinsulinemia, dyslipidemia and hyperglycemia [4], while the effect of MSx-induced hyperinsulinemia and other related parameters on the IGFs/IGFBPs elements has not been fully understood [4, 9–11]. In this study, we investigated the impact of MSx on serum levels of IGF-1 and IGF-2 along with their binding proteins, namely IGFBP-1, -2, and -3. Moreover, since the components of the IGF axis would be at the zenith of their concentrations during adolescence [4], adolescent patients with MSx are seemingly eligible to be examined for the evaluation of obesity effect on the levels of the components of IGFs/IGFBPs and their relationship with the pathogenesis of MSx.

## Main text

### Methods

A total of 220 cases were selected from individuals who referred to the endocrinology ward of Imam Khomeini Hospital in Urmia, between May 2015 and September 2017, and then categorized into two groups: metabolic syndrome x group that consisted of 65 men and 45 women with the mean age of 45.8 ± 9.8 years and the control group, including 65 men and 45 women with mean age of 42.8 ± 13.1 years. The healthy individuals (control group) were age- and sex-matched. None of the healthy subjects had a history or any signs of metabolic syndrome. Patients were included in the metabolic syndrome x group if they met three out of five inclusion criteria as follows: central obesity (waist circumference > 102 cm in men and > 88 cm in women), having a low concentration of high-density lipoprotein (HDL) cholesterol (< 40 mg/dl in men and < 50 mg/dl in women), hypertriglyceridemia (triglycerides > 150 mg/dl), and hypertension (> 130/85 mmHg). The exclusion criteria were having systemic diseases, and previous use of antilipidemic, antidiabetic, antihypertensive, contraceptive, and diuretic drugs. Pregnant individuals and patients with diabetes, inflammation, and infection were excluded as well. Written informed consent was obtained from all participants before they took part in our study.

The demographic characteristics of all study subjects, including sex, age, systolic and diastolic blood pressure, waist circumference, body weight, and body mass index (BMI) were also recorded. There was no difference between the two groups when smoking was statistically considered (data not shown). Fasting venous blood samples were collected from each participant. Serum glucose and lipid profile (triglycerides, total cholesterol, HDL-C, and LDL-C) were determined by the spectrophotometry method (Pars Azmoon, Iran). Serum insulin was measured using an enzyme-linked immunosorbent assay (ELISA) (Abcam, United Kingdom).

#### Measurement of serum IGF-1, IGF-2, IGFBP-1, IGFBP-2, IGFBP-3

According to manufacturers’ instructions, the quantitative analysis of serum IGF-1 (Abcam, UK) IGF-2 (ALPCO, USA), IGFBP2 (Abcam, UK), IGFBP2 (Abcam, UK) and IGFBP-3 (Abcam, UK), were performed utilizing the ELISA Kits.

#### Statistical analysis

The statistical analysis was carried out by the SPSS software version 15.0 (SPSS Inc., Chicago, IL). One-way analysis of variance (ANOVA) was applied for multiple comparisons among groups followed by Tukey’s post hoc test. Also, for the comparison of the values between subgroups, the unpaired Student T-test was used. Pearson correlation coefficient was utilized for the assessment of the association between variables. The level of statistical significance was accepted if the p-value was less than 0.05.

### Results

The demographic characteristics and biochemical parameters of all individuals are presented in Table 1. As shown in Table 1, the demographic variables (BMI and waist circumference) and clinical parameters (serum total cholesterol, HDL cholesterol, LDL cholesterol, and triglyceride) show a significant difference between the control and MSx groups. Moreover, insulin serum level was significantly (p < 0.05) higher in the MSx group compared with the control group.

**Table 1:** Demographic and biochemical features of studied population

Parameters	MS	Controls	p value
Age (year)	45.8 ± 9.8	42.8 ± 13.1	> 0.05
BMI (kg/m^2^)	4.5 ± 34.1	5.6 ± 25.6	< 0.001
WC (cm)	8.8 ± 116.1	9.59 ± 89.2	< 0.001
SBP (mmHg)	9.5 ± 130.5	10.2 ± 128	> 0.001
DBP (mmHg)	6.9 ± 86.8	7.1 ± 75.1	> 0.001
FBS (mg/dl)	19.6 ± 115.4	9.8 ± 86.2	< 0.001
TG (mg/dl)	122.4 ± 250.3	38 ± 114.6	< 0.001
TC (mg/dl)	101.3 ± 252.9	28.9 ± 148	< 0.001
HDL-C (mg/dl)	10.2 ± 52.2	8.3 ± 55.8	< 0.05
LDL-C (mg/dl)	30.2 ± 135.1	11.9 ± 95.1	< 0.001
Insulin (pmol/l)	9.2 ± 109	13.4 ± 69.1	< 0.001

Data are presented as mean ± standard deviation

*MS* metabolic syndrome X, *WC* waist circumference, *SBP* systolic blood pressure, *DBP* diastolic blood pressure, *TC* total cholesterol, *LDL*-*C* low-density lipoprotein cholesterol, *HDL*-*C* high-density lipoprotein cholesterol, *TG* triglycerides

As displayed in Table 2, the serum levels of IGF-1, IGFBP-1, and IGFBP-2 were markedly decreased in patients with MSx compared with those in the control group (p < 0.05). When the concentrations of IGFBP-3 and IGF-2 were compared between the two groups, the MSx group had a lower level of each factor in comparison with the control group; however, the difference between the two experimental groups was not statistically significant (p > 0.05). The results showed a significant difference in the concentrations of IGF-1, IGFBP-1, and IGFBP-2 between the MSx and control groups. Patients with MSx had lower levels of IGF-1, IGFBP-1, and IGFBP-2 compared with healthy individuals (p < 0.05). Although, the concentrations of IGFBP-3 and IGF-II were diminished in the MSx group in comparison with the control group; such a decrease was not statistically significant (p > 0.05).

**Table 2:** Serum IGFs/IGFBPs levels in MS and controls

Parameters	MS	Controls	p value
IGF-1 (ng/ml)	20 ± 2.6	29 ± 4.3	> 0.05
IGF-2 (ng/ml)	3.9 ± 1.8	4.5 ± 3.3	<0.05
IGFBP-I (pg/ml)	398.5 ± 62.5	482.5 ± 73.5	<0.05
IGFBP-2 (pg/ml)	829.3 ± 95.3	846.8 ± 89.2	> 0.05
IGFBP-3 (pg/ml)	699.6 ± 56.3	714.9 ± 46.7	> 0.05

Data presented as mean ± SD, p < 0.05 considered as significant level

*IGF*-*1* insulin-like growth factor-1, *IGF*-*2* insulin-like growth factor-2, *IGFBP*-*1* insulin-like growth factor binding protein-1, *IGFBP*-*2* insulin-like growth factor binding protein-2, *IGFBP*-*3*: insulin-like growth factor binding protein-3, *MS* metabolic syndrome

#### Correlation analysis

The correlation analysis of examined parameters demonstrated a significant positive correlation between the serum levels of IGF-1 and IGF-2 (p < 0.05). Similarly, there was a significant relationship between the serum concentrations of IGFBP-3 and IGF-2 (p < 0.05). The statistical analysis also showed a negative association between the serum levels of IGF-1 and plasma glucose and total cholesterol levels (p < 0.05). Also, a significantly positive correlation was found between the age and serum concentrations of IGF-I/IGF-2 (p < 0.05).

### Discussion

IGFs, their IGFBPs, and their cognate receptors have essential roles in growth and metabolism. Also, the IGF system plays a significant role in pathophysiological events in the development of insulin resistance and the emergence of its complications [12, 13]. Consequently, IGF and its binding proteins are thought to be altered during the pathogenesis of MSx, usually accompanied by insulin resistance. Hence, in the present study, we evaluated the concentrations of IGF/IGFBP components in patients with MSx and their relationship with clinical parameters of the disease.

As shown in Table 1, MSx patients had lower levels of IGF-1, IGFBP-1, and IGFBP-2 compared with healthy subjects. Notably, compared with the control group, the levels of IGFBP-3 and IGF-2 were reduced in the MSx groups; however, such a reduction was not statistically meaningful. In a study performed by Attia and colleagues, they assessed the regulation of the IGF components in overweight adolescents. Consistent with our findings, they reported that obese adolescents with elevated levels of insulin had lower concentrations of total IGF-1 and IGFBP-2 compared with their normal-weight counterparts. It implies that these finding may be due to hyperinsulinemia that could result in a decrease in the level of IGFBP-1. A reduction in concentrations of IGFBP-1 in hyperinsulinemia condition might stem from higher bioavailability of the free IGF-1, as well as the decreased levels of total IGF-1 and IGFBP-3 [4]. The results of our study were in agreement with these findings.

In another study conducted by Helad et al., they measured fasting levels of IGFBP-2, IGFBP-1, IGFBP-3, IGF-1, and IGF-2 in diabetic patients who were assigned to two groups: MSx diabetic and non-MSx diabetic patients. The authors indicated that MSx patients had a lower concentration of IGFBP-2 compared with healthy subjects. They revealed an inverse correlation between IGFBP-2 and blood glucose as the low levels of IGFBP-2 was associated with elevated fasting glucose. They also showed a positive relationship between the concentration of IGFBP-2 and development of MSx. Correspondingly, decreased levels of IGFBP-2 and IGFBP-1 were correlated with the risk of cardiovascular disorders [12]. The results of our study showed a negative correlation of serum IGF-1 with plasma glucose and total cholesterol levels (MSx-related parameters). According to the obtained results, there was a decline in IGF-1 level that was statistically associated with higher levels of glucose and the development of dyslipidemia in our patients.

It is thought that a decrease in IGFBP-1 level is likely owing to the elevated insulin concentrations, which lead to the disturbance in IGFBP-1 regulation. This phenomenon is caused by an inverse regulatory effect of free IGF availability. It seems that IGFBP-1 level is conversely linked to the concentration of insulin [13, 14]. Based on our findings, we observed a negative correlation of serum IGF-1 with that of plasma glucose and total cholesterol levels in MSx patients. Previous studies have shown that IGF-1 levels were associated with cardiovascular complications such as insulin resistance, MSx, and also lipid concentrations [5, 14–19]. In line with our results, a recent study showed a converse connection between the elevated levels of IGF-1 and total cholesterol, [20]. Similar to our findings, another study showed a positive linkage of LDL-c cholesterol with IGF-1 and IGFBP-3 concentrations [15].

Additionally, in our survey, there was a positive correlation between serum IGF-1 level and IGF-2 (p < 0.05), serum IGFBP-3 and IGF-2 (p < 0.05), and between the age of participants with serum IGF-1 and IGF-2 levels. These findings support the notion that alterations in the regulation of some elements of the IGF axis can occur during the pathogenesis of MSx. Our findings revealed that MSx is associated with perturbations in the IGF/IGFBP system when assayed in fasting state. Similar to the results obtained from the study performed by Attia et al., we indicated that decreased circulating level of total IGF-1 is considerably associated with declined concentrations of IGFBP-1, IGFBP-2, and IGFBP-3 [4]. The role(s) of the IGF/IGFBP system in the modulation of glucose and lipid metabolism would be pronounced if the balance of the components of the IGF/IGFBP system is impaired; however, the mechanism underlying this regulation has not been recognized yet [21]. It is now known that there is a defect in the modulation of the IGF/IGFBP system during MSx pathology. It could be inferred that the elements of the IGF/IGFBP system, as the vital constitutes of the system, play an indispensable role in the development of MSx. Nonetheless, future studies with the aid of protein and gene expression analyses, and large sample size will shed light on our understanding of the impact of the IGF/IGFBP system on the development and pathology of MSx.

## Limitations

Although the current study successfully indicated that some components of IGFs/IGFBPs are associated with the risk of MSx, due to the insufficient budget we were not capable of performing further analyses at the level of gene expression, western blot, and ELISA. Further studies are needed to find out the effects of genetic components on the above-measured factors.

## Data Availability

The datasets analyzed during the current study are available from the corresponding author on reasonable request.
